# The impact of snacking habits and physical activity on body composition in overweight and obese adolescents: A longitudinal study differentiating home and school environments

**DOI:** 10.1371/journal.pone.0318000

**Published:** 2025-02-26

**Authors:** Xiang Pan, Yibo Gao, Yanfeng Zhang, Koya Suzuki, Xiaoxiao Chen, Jin He, Xueli Zhao, Lupei Jiang, Aoyu Zhang, Yibei Wang, Deqiang Zhao

**Affiliations:** 1 China Institute of Sport Science, Beijing, China; 2 Graduate School of Health and Sports Science, Juntendo University, Inzai, Japan; 3 Juntendo Administration for Sports Health and Medical Sciences, Juntendo University, Tokyo, Japan; 4 College of Physical Education and Sports Rehabilitation, Jinzhou Medical University, Jinzhou, China; 5 Department of Psychology, Qingdao University, Qingdao, China; University of Glasgow School of Health and Wellbeing, INDIA

## Abstract

With rising incomes in developing countries, the required necessary physical activity declines and the availability of snacks increases, further causing obesity in adolescents. The study was a longitudinal two-stage observational study of 74 overweight and obesity adolescents distinguishing between school and home environment phases. Data were collected at three time points (mid-semester (T0), end of semester (T1), and end of the winter holidays (T2)), and explorations were conducted using multivariate linear regression and Granger causality tests to investigate how changes in moderate-to-vigorous physical activity(MVPA), snacking habits (proportion of snack calorie, PSC; proportion of snack calories from protein, PSCP) in multiple stages and how their changes affect body composition. The results showed that during the semester phase, increases in ΔMVPA and ΔPSCP and decreases in ΔPSC were associated with decreases in ΔBFP (Δ = post-timepoint value − pre-timepoint value). During the holiday phase, decreases in ΔPSCP, MVPA (T1), and PSCP (T1), and increases in ΔPSC were associated with increases in ΔBFP. Only MVPA having a positive predictive effect causally on FFM. Snacking habits have a significant impact on body composition changes in adolescents, and reducing snack intake and choosing high-protein snacks are critical to controlling obesity in adolescents, especially during the holiday period. Strategies to increase MVPA should also be implemented to increase FFM briefly indirectly control obesity.

## 1. Introduction

Snacking is more popular among children and adolescents in developed countries [1], but with rapid economic development, the diversity of snacks has begun to diversify in some developing countries, with up to 75% of Asian children starting to consume sugary snacks at the age of two years [2], and much of the current research focuses on snack intake in low- and middle-income populations [2,3].

The situation in China is slightly different, though, as studies from nearly two decades ago concluded that Chinese children have a much lower proportion of snack calories (PSC) to daily dietary energy [4]. However, China’s gross domestic production per capita has rapidly increased from less than USD 1,000 at the beginning of the 21st century to more than USD 12,000 in 2023 [5], classifying it as an upper-middle-income country. Along with the highly advanced e-commercial industry, the market size of China’s snacks industry grew from 822.4 billion yuan to 1298.4 billion yuan from 2016 to 2020 [6], and the top three categories such as candy preserves, nuts, and fried food, puffed food accounted for more than 80%, all of which are high-sugar and high-oil snacks [7], and Chinese children often choose those snacks because they are tasty [8]. The growth in PSC seems to bring great obesity potential, the proportion of snack calories is positively correlated with obesity [9], and the high frequency of snack intake is related to the prevalence of overweight and obesity in children and adolescents [10]. Chinese scholars analyzed the data from China Health and Nutrition Survey in 1997, 2000, 2004, 2006, and 2009, and found that the PSC and the body mass index (BMI) were on the rise for young women and that the risk of obesity was higher for those with a PSC of more than 10% [11].

Although the negative effects of snacking cannot be avoided, higher protein seems to be a good choice as high protein diets are beneficial in improving body composition [12], and glycemic control [13]. High-protein, low-carbohydrate snacks significantly reduced fasting blood glucose, insulin, and nighttime blood glucose levels and improved insulin sensitivity index than high-carbohydrate snacks [14]. Furthermore, low frequency of physical activity is associated with the prevalence of overweight and obesity in children and adolescents [10], PA and BFP are negatively correlated [15,16], and exercise training in the context of strict interventions can counteract the increase in BFP caused by small amounts of high oil and high sugar snacks intake [17], However, different environments seem to lead to larger differences, such as snack intake [8] and PA [18] not being the same during school and holiday periods.

While some studies have shown that snack intake is strongly associated with obesity [10,11,25], most of these studies have used cross-sectional designs and there are relatively few studies of children from high-income families in developing countries, and in particular, there is a lack of cohort studies of the proportion of snack intake and its protein share in relation to changes in body composition across different life situations (e.g., during school vs. during vacation). We therefore asked two research questions: How do PSC, PSCP, and physical activity change during school and holiday for middle school students with High-Family-Income? Are changes in PSC, PSCP, and physical activity associated with body composition changes? These correspond to the purpose of our study. The initial goal was to compare the stage changes in PSC, proportion of snack calories from protein (PSCP), and Moderate- to high-intensity physical activity (MVPA) and body composition between the two stages. The further goal was to explore the dynamic relationship between the phase changes of PSC, PSCP, and MVPA and the changes in body composition, and to further analyze the causal pathways.

## 2. Methods

### 2.1. Subject screening

**Fig 1:**
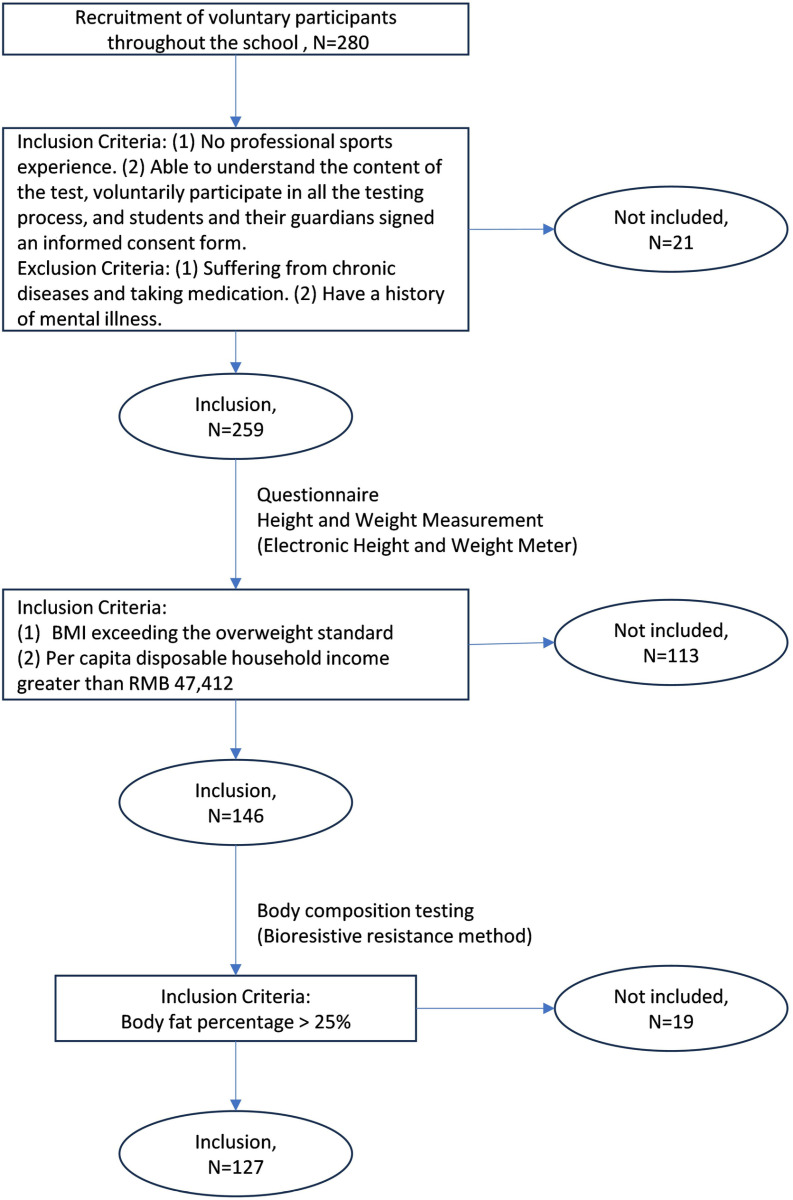
Recruitment and screening flowchart.

PASS 15.0 was used to calculate sample size, and since the study involved multiple linear regression with change in the independent variable indicator in the follow-up, and the amount of change as a variable, the sample size was calculated separately and then the minimum sample size to satisfy the higher was used for recruitment. Power = 0.9, Alpha = 0.15, variable R^2^ = 0.2, covariate R = 0.1, and the number of variables totaled 9. A minimum sample size of 53 was calculated to recruit as many study subjects as possible, taking into account the potential for significant attrition in a multi-stage follow-up test and the high motivation of the subjects. Recruitment took place from September 10 to October 7, 2023. A four-stage recruitment and screening process was used. The recruitment and screening process is illustrated in Fig 1. Figure Notes: First excluding ineligible volunteers from the 280 volunteers recruited. Inclusion criteria: (1) no professional sports experience; (2) ability to understand the content of the test, voluntary participation in the full testing process, and informed consent signed by students and their guardians. (3) no chronic illness or taking medication; (4) no history of mental illness. After screening, a total of 259 individuals entered the next stage with questionnaires and electronic height and weight measurements (electronic height tester GMCS-SGJ3, electronic weight tester GMCS-RCS3), with inclusion criteria: individuals with per capita household disposable income >47,412 yuan [19] and with a BMI exceeding the criteria for overweight [20]. Finally, 146 individuals were screened for body composition testing (bioimpedance method, Inbody 3.0), inclusion criteria: subjects with BFP > 25% [21] to avoid high BMI but low BFP, and 127 subjects meeting the study requirements were included.

### 2.2. Procedure

A longitudinal cohort study design was used to follow up the 127 students included in the study for a period of 18 (2 weeks for the test week and 16 weeks for the interval week). To ensure transparency and reproducibility of our findings, we adhered strictly to STROBE guidelines for study design and reporting. The STROBE flowchart (S1 Fig) and STROBE checklist (S1 Table) illustrate the detailed steps and content of our study. Data were collected three times in the middle of the semester (T0), at the end of the semester (T1), and at the end of the winter holiday (T2), and each data collection period was 4 days with an interval of 8 weeks, with the Q1 phase between T0 and T1, and the Q2 phase between T1 and T2, which was natural without any intervention from the study. The study site was a secondary school in Beijing, longitude: 116.31 latitude: 39.77, northern China, with Q1 in autumn (during the semester at school) and Q2 in winter (during the winter holiday). The study was approved by the Experimental Ethics Committee of China Institute of Sport Science (protocol code CISSLA-20221116).

### 2.3. Test methods

#### 2.3.1. Morphological indicators.

Height and weight were measured using an electronic height tester (model GMCS-SGJ3, Beijing, China) and a weight tester (model GMCS-RCS3, Beijing, China). BFP, FFM, and BF were measured using the bioimpedance method (Body Composition Tester, Inbody 3.0, Korea).

#### 2.3.2. Dietary status.

Parents of the subjects were trained to complete a four-day, 24-hour dietary review questionnaire, which included three schoolling days and one nonschoolling day. Additionally, we specifically requested that regular meals and snacks be labeled. To optimize the questionnaire and reduce dietary recall bias, we have added pictures of common food items and portion reference charts to help subjects and their guardians recall and estimate food types and intake more accurately. In addition, the questionnaire adopted a more structured design to guide subjects to recall more details, such as cooking methods and seasonings added, to reduce omissions. Finally, we asked subjects to record multiple times in a day to obtain more comprehensive and accurate dietary information. Dietary records and nutrient intake calculations will be performed simultaneously using the Boohee APP (Shanghai, China) as a second data recording to correct the accuracy of the data, and if there is a difference of more than 10% of the original value, the final value will be calibrated again after a face-to-face communication with the participant for checking. These data will be used to analyze the subjects’ dietary habits. PSC =  (sum of calories intake from snacks outside the three main meals)/(total calories intake for the whole day) *  100%; PSCP =  (calories intake from protein/total calories intake from snacks outside of the three main meals) *  100%. Daily averages were calculated separately and valid values were calculated as [(weekday average *  5) +  (weekend average *  2)]/7.

#### 2.3.3. Physical activity.

Moderate-to-vigorous physical activity (MVPA) is defined as physical activity performed at a moderate or higher level of intensity. The effectiveness of three-axis accelerometer has been validated in many studies [22,23]. In this study, the Actigraph GT3X+ accelerometer was used to assess MVPA in subjects, who were instructed to wear the accelerometer in their left hand throughout the day and record it for at least four days including at least one day on the weekend. The sampling frequency is 30 Hz, the sampling interval is 1 min/epoch. A day was excluded from the analysis if the signal was not detected for more than one hour or the daily Counts do not exceed 20,000 CPM. MVPA was calculated using the formula [(weekday average *  5) +  (weekend average *  2)]/7.

### 2.4. Method of analysis

In this study, SPSS26.0 was used to statistically analyze the subjects’ data. The Shapiro-Wilk test was used to test for normal distribution of the data at T0, T1, and T2 respectively, and the paired samples t-test (for normal distribution) or the paired samples wilcoxon signed-rank test (for non-normal distribution) was used to analyze the variability of the three data, involving a two-by-two comparison of three components, and the Bonferroni correction was used to adjust the significance level to control for Type I errors, with the Bonferroni correction giving a significance level of 0.05/3 =  0.0167 and a highly significant level of 0.01/3 = 0.0033. After the Shapiro-Wilk test for normal distribution of the data for each indicator of ΔQ1 and ΔQ2, a paired samples wilcoxon signed rank test was performed with a significance level of 0.05 and a highly significant level of 0.01, and the correlation between the amount of change in each indicator in the Q1 and Q2 phases was analyzed using the Spearman’s rank correlation coefficient, respectively. Multiple linear regression models were used and the degree of model fit and statistical significance were assessed to explore the effect of multiple factors. Granger causality tests were performed based on the Python 3.12 environment [24].

## 3. Results

### 3.1. Descriptive statistics

As shown in Table 1, a total of 127 subjects were recruited for this study. 53 were lost during the follow-up period due to school transfer, suspension, voluntary withdrawal, refusal to participate in the follow-up test, unavailability of data, etc., resulting in a final valid sample size of 74 subjects aged between 13 and 15 years, with 43 males (58.11%) and 31 females (41.89%). Descriptive statistics for each test indicator are shown in Table 1, with average PSCP at only 11.6% and average PCS as high as 33.0%.

**Table 1:** Descriptive statistics.

Variable	Mean±SD or N (%)
**Age (years)**	
**13**	27 (36.49%)
**14**	28 (37.84%)
**15**	19 (25.68%)
**Gender**	
**Male**	43 (58.11%)
**Female**	31 (41.89%)
**Height (cm)**	164.3 ± 8.05
**Weight (kg)**	60.9 ± 14.50
**FFM (kg)**	44.3 ± 9.51
**BF (kg)**	17.6 ± 5.82
**BFP (%)**	29.2 ± 3.91
**MVPA (min/d)**	53.3 ± 5.96
**PSC (%)**	33.0 ± 7.45
**PSCP (%)**	11.6 ± 2.29

FFM, fat free mass; BF, body fat mass; BFP, body fat percentage; MVPA, moderate-to-vigorous physical activity; PSC, proportion of snack calorie; PSCP, proportion of snack calories from protein.

### 3.2. Multi-stage changes in body composition, PSC, PSCP, MVPA

Between-group differences were analyzed and described for the three times indicators and their changes in volume, expressed as mean ±  standard deviation for those with normal distribution and P50 (P25, P75) for those with non-normal distribution, as detailed in Table 2. Height increased significantly in both Q1 and Q2 phases, with ΔHeight (Q1) being higher than ΔHeight (Q2). The weight change was not significant in Q1 phase and increased significantly in the Q2 phase, with a median Δweight (Q2) of 0.5 kg.

**Table 2:** Changes in body composition, PSC, PSCP, MVPA.

Variable	T0	T1	T2	ΔQ1	ΔQ2
**Height (cm)**	164.3 ± 8.05	164.6 ± 8.04aa	164.8 ± 8.04aabb	0.20 (0.10, 0.30)	0.10 (0.10, 0.20)cc
**Weight (kg)**	60.9 (54.6, 73.5)	60.95 (53.8, 73.3)	62.0 (54.9, 74.7)aab	0.55 (−1.60, 0.90)	0.50 (0.38, 1.50)cc
**BFP (%)**	29.2 ± 3.91	28.4 ± 4.06aa	29.4 ± 3.80bb	−0.20 (−2.45, 0.63)	0.30 (0.20, 1.80)cc
**FFM (kg)**	44.3 (39.6, 50.9)	44.6 (39.6, 51.5)aa	44.7 (39.7, 51.6)abb	0.20 (0.10, 0.50)	0.10 (0, 0.20)cc
**BF (kg)**	17.6 (15.0, 23.3)	17.3 (14.1, 22.6)	17.9 (15.0, 23.9)aab	0.10 (−1.85, 0.60)	0.35 (0.20, 1.43)cc
**PSC(%)**	42.2 (36.1, 45.4)	33.2 (32.1, 40.0)aa	37.0 (32.8, 45.4)	−7.15 (−12.30, −1.50)	7.80 (0, 10.32)cc
**PSCP (%)**	11.6 (10.0, 13.4)	13.75 (11.4, 15.9)aa	13.1 (11.0, 15.4)abb	1.85 (0.29, 3.93)	−0.65 (-1.15, −0.35)cc
**MVPA (min/d)**	53.3 ± 5.96	53.6 ± 7.91	53.7 ± 8.58	−0.36 (−2.26, 2.72)	−0.08 (−0.48, 1.38)
**BFP (%)**	29.2 ± 3.91	28.4 ± 4.06aa	29.4 ± 3.80bb	−0.20 (−2.45, 0.63)	0.30 (0.20, 1.80)cc

a indicates the P-value of the difference between T1 and T2 compared to T0, a: < 0.05, aa: < 0.01.

b indicates the P-value of the difference between T2 compared to T1, b: < 0.05, bb: < 0.01.

c indicates the P-value of the difference between ΔQ1 compared to ΔQ2, c: < 0.05, cc: < 0.01.

ΔQ1 is the amount of change during the Q1 phase, i.e., T1 − T0.

ΔQ2 is the amount of change during the Q2 phase, i.e., T2 − T1.

Both height and weight increased more in Q1 than in Q2 (P <  0.05), and BFP decreased and then increased in both phases, with no significant change in BF but an increase in FFM of 0.2 KG (P <  0.0033) in Q1, and a median ΔBFP (Q1) of −0.2%, while BF increased by 0.35 kg in Q2, and FFM increased by 0.10 kg, with a median ΔBFP (Q2) of 0.3% (p <  0.0033). PSC decreased by 7.15% (P <  0.0033) in Q1 and rebounded slightly in Q2, with a non-significant difference, and PSCP had a median value added of 1.85% (P <  0.0033) in Q1 and decreased by 0.65% (P <  0.0033) in Q2. MVPA did not show a significant change in any of the three tests.

### 3.3. Influencing factor model for BFP

Distinguishing the stages of Q1 and Q2, the correlation of each indicator with ΔBFP and ΔFFM was calculated separately, and after excluding the covariate indicators, the regression models of ΔBFP and ΔFFM in Q1 and Q2 stages were established by adjusting and fitting several times.

As shown in Table 3, the adjusted R-squared of the ΔBFP influencing factor model reached 0.781 in Q1, and the overall explanatory power of the model was good. ΔMVPA (Q1) with a β value (unstandardized coefficient) of −0.494 (P = 0.000), ΔPSCP (Q1) with a β value of −0.226 (P = 0.009), and as ΔMVPA and ΔPSCP increased, there was a negative association of ΔBFP (Q1). ΔPSC (Q1) with a β value of 0.528 (P = 0.000), with increasing ΔPSC, ΔBFP (Q1) was positively associated. In addition, MVPA(T0) had a β value of −0.122 (P = 0.046), and taking into account the other variables, an increase in MVPA(T0) was negatively associated with a decrease in ΔBFP(Q1), albeit with a low effect size.

**Table 3:** Factors influencing body fat percentage at Q1.

Variable	B	SE	β	P	VIF
**ΔMVPA(Q1)**	−0.267	0.041	−0.494	0.000	1.945
**MVPA(T0)**	−0.04	0.02	−0.122	0.046	1.207
**ΔPSC (Q1)**	0.152	0.022	0.528	0.000	2.026
**ΔPSCP (Q1)**	−0.177	0.066	−0.226	0.009	2.353

Tip: B =  Regression Coefficient; SE =  Standard Error; β =  Standardized Regression Coefficient; P =  P-value; VIF =  Variance Inflation Factor.

As shown in Table 4, the adjusted R-squared of the model of ΔBFP influencing factors reached 0.707 at the Q2 stage, with a good overall explanation of the model. ΔPSCP (Q2) had a β value of −0.508 (P = 0.000), and there was a negative correlation of ΔBFP (Q2) as ΔPSCP (Q2) increased. The rank order of the weights of the factors that showed positive associations was MVPA (T1) (β = 0.632, P = 0.000), PSC (T1) (β = 0.513, P = 0.000, and ΔPSC (β = 0.218, P = 0.001). This indicates that as these variables increased, ΔBFP (Q2) also tended to increase.

**Table 4:** Factors influencing body fat percentage at Q2.

Variable	B	SE	β	P	VIF
**ΔPSCP (Q2)**	−0.851	0.116	−0.508	0.000	1.327
**ΔPSC (Q2)**	0.043	0.012	0.218	0.001	1.099
**MVPA (T1)**	0.09	0.015	0.632	0.000	3.072
**PSC (T1)**	0.077	0.015	0.513	0.000	2.739

### 3.4. Influence factor model for fat-free mass

As shown in Table 5, adjustment R^2^ =  0.277 for the ΔFFM (Q1) model, which changes only associated with PSCP (T0) and BFP (T0), PSCP (T0) (β = 0.549, P =  0.000) and higher PSCP (T0) positively influenced ΔFFM (Q1), BFP (T0) (β =  0.446, P =  0.000) and higher BFP (T0) positively influenced ΔFFM (Q1). No indicator entered the model for ΔFFM (Q2).

**Table 5:** Factors influencing FFM at Q1.

Variable	B	SE	β	P	VIF
**PSCP(T0)**	0.032	0.008	0.446	0.000	1.209
**BFP(T0)**	0.016	0.003	0.549	0.000	1.199

### 3.5. Causal paths

As shown in Table 6, for the change in BFP, no significant path was found or showed inverse time causality, whereas for the change in FFM, in stages Q1 and Q2, FFM (T1) and FFM (T2) were significantly influenced by MVPA at the previous time nodes, MVPA (T0) (P = 0.035) and MVPA (T1) (P = 0.034), respectively. FFM (T0) was significantly in-fluenced on FFM (T2) (P = 0.028).

**Table 6:** Causal paths for FFM.

Independent Variable	Dependent Variable	P
–	FFM(T0)	–
MVPA(T0)	FFM(T1)	0.035
FFM(T0)	FFM(T2)	0.028
MVPA(T1)	FFM(T2)	0.034

## 4. Discussion

In our study, higher changes in PSC were positively associated with higher changes in BFP. It is well-researched that snack intake is highly associated with obesity [9–11,25]. A study of a middle-aged Danish population found that eating one additional regular meal per day was associated with a decrease in BMI over the following 6 years, with similar negative trends for BF, FFM, and waist circumference, whereas PSC was positively associated with an increase in BFP, and that regular meal intake, rather than frequent snack intake, may be helpful in controlling weight and BFP growth [25]. Scholars in China investigated the relationship between snack consumption and changes in BFP in 2368 children aged 6–14 years and found that high snack consumption was significantly associated with a rapid increase in BFP [26]. High-calorie diets, especially frequent intake of high-fat and high-sugar snacks, can lead to decreased serotonin transporter binding activity in hypothalamic regions. Reduced serotonin transporter, in turn, may lead to elevated serotonin levels in the synaptic gap, which in turn affects hypothalamic function and down-stream neurotransmission, leading to problems with mood regulation, increased appetite, and decreased satiety, thus increasing the risk of overeating and weight gain [27]. Even if it does not directly contribute to obesity, some unhealthy snacks may have a negative impact on health. For example, studies have shown that donut intake may impede the improvement of vascular endothelial function by exercise [17], or negatively affect glycolipid metabolism and quality of life in diabetic patients [28].

However, some studies did not find a direct association between snack intake and obesity, for example, Feifei He et al. based on a Chinese database also did not find an association between snack consumption and the risk of overweight and obesity in Chinese children and adolescents [29], but the PSC of the survey participants was 2.4%, which is quite different from the obese samples in our study, and the variability of the study samples might have also led to the effect of PSC on BFP, for example, studies of sample income and snack choices [8], while healthy snacks may contribute to weight control [14]. Animal studies have also shown that obesity can be avoided by selectively controlling the intake of high-fat and high-sugar foods [30]. This also coincides with a finding in our study that the negative effects on body composition can be attenuated with a higher percentage of protein in snacks. We found that high PSCP was associated with increased FFM and that increased PSCP was associated with decreased BFP. Several studies have shown that increasing protein intake increases FFM versus decreasing BF [1,7,13] and improves body composition even when weight is stable [31]. Protein causes more satiety than carbohydrates and fat [32]. High-protein diets can affect appetite and satiety through many mechanisms, including increasing energy expenditure, modulating hormone secretion, and influencing amino acid levels with a higher thermic effect [32]. The source of protein and how it is eaten may also influence its effect on body composition, such as increasing FFM through beef supplementation, which needs to be combined with a higher daily protein intake and correct supplement time [33]. However, frequent intake of high-fat and high-sugar snacks, even if high in protein, may negatively affect brain function and increase the risk of obesity and other health problems [27].

Furthermore, the place and time of snack intake may also be related to its association with obesity, as our study showed that children’s PSC increased during the holiday period and that BFP changes were influenced by PSC changes to a higher degree. This can be rationalized by the fact that in school snacks are less available due to management from teachers, many curricula, etc. It is interesting to note that within the school where we implemented the study, there was only one shop selling extremely small assortment of snacks and there was only one convenience store selling snacks within 1km of the school, which may have contributed to the lower PSC of the children during the school day. Whereas home is the primary place where snacks are available to children [8], during the holidays, parents often provide snacks as a reward and to celebrate the holiday [3]. In addition, the winter holidays include the most important Chinese festival, the Spring Festival, and perhaps children’s eating habits are temporarily affected by the festival; after all, most observational studies have found that weight gain occurs during the holiday season [34]. This suggests that parents need to better manage their children’s snack intake during the holidays.

In summary, high PSC and low PSCP are associated with increased BFP, which again demonstrates the established link between a high sugar and high fat diet and obesity. This suggests that obesity management in Chinese adolescents from high-income families should focus on the risk of fat gain associated with excessively high PSC, and on healthier snack choices that include a higher proportion of protein and lower levels of sugar and fat. Moreover, schools and families should work together to reduce the availability of snacks, especially parents in the family setting should try to avoid children’s access to snacks and manage snack intake during holidays to prevent their overweight and obesity from increasing.

MVPA was associated with a reduction in BFP at Q 1. The relationship between MVPA and optimization of body composition has been well researched [16,33,35–37] confirming that exercise training interventions to increase MVPA can be effective in lowering BFP, however, in our case we found no causal relationship between the two, possibly due to the obesity-specific nature of the study sample itself as well as the effect of the absence of any dietary guidance interventions. After all, exercise interventions alone without combining with calorie restriction have very little effect on body composition [38]. In addition, we found that MVPA has a causal effect on FFM and can predict changes in FFM. Therefore, we believe that the reason why the BFP decreased with MVPA increasing was due to the increase of FFM and that it could counteract the BFP change due to the PSC elevation in the current study. Another study [34] also stated that although low calorie diets are commonly used to reduce BFP, this strategy is only effective in the short term, and that high intensity exercise best promotes BFP reduction to achieve medium to long term health goals. Therefore in situations where long-term sustained calorie control or natural state is not possible, increasing MVPA should be a powerful means of increasing FFM. Regardless of the school and home environment, administrators or guardians should increase MVPA in overweight and obese adolescents to promote a decrease in BFP or to control only the stabilization of it.

By studying the high-income group in developing countries, which is more specific in terms of socio-economic status, lifestyle, and health risks, we can gain insight into their health status, behavioral patterns, and needs. This fills a gap in previous research on cohort studies of the relationship between snack intake and body composition in adolescents from high-income families in developing countries. The study focuses on the specific context, i.e., the school environment and the home environment during holidays, making it easier to analyze the situation in depth in that context. By collecting data at three-time nodes, the study was able to observe trends in students’ body composition, dietary habits, and physical activity, and explore the interrelationships among these factors, which revealed more causality and developmental pathways than a cross-sectional study. The effect of the proportion of protein in snack intake was analyzed, which provides a new perspective for studying the effects of snacking on body composition. The use of Granger causality tests allows for a deeper exploration of influences and causality among multiple factors beyond simple correlation analyses, increasing the reliability and persuasiveness of the findings. However, it is possible that the presence of a policy intervention (a national policy to increase extracurricular physical activity) factor during the study period resulted in higher levels of MVPA, which may limit the generalizability of the impact study results and the replication in other countries and regions.. Sample attrition is a common problem in longitudinal studies, and the sample attrition in this study was a bit larger, although still above the minimum required sample size, and may still be in a way that slightly reduces the statistical effect. In addition, the sample in this study was only drawn from children in high-income families in Beijing, and the findings may not be directly generalizable to children in families with other income levels; therefore, caution is needed when generalizing the results of this study to other populations or contexts. Larger and longer follow-up studies are needed in the future to validate our findings.

## 5. Conclusions

In the school environment where the availability of snacks is low, PSC decreases, FFM increases, and BFP decreases; while in the family environment during the vacation, PSC increases and PSCP decreases, FFM and BFP increase, and there is no significant change in MVPA in both environments. The increase in PFP is related to the increase in PSC, and the decrease in PSCP, but not related to the change in MVPA. The increase in FFM is affected by the increase in MVPA. In conclusion, snacking habits and protein intake significantly influence changes in body composition among overweight and obese secondary school students. Reducing snack intake, choosing high-protein snacks and increasing physical activity, especially during holidays, are essential for managing adolescent obesity.

## Supporting information

S1 Fig STROBE flowchart. (JPG)

S1 Table STROBE checklist. (PDF)
