# Supplementary figures and images for: The impact of snacking habits and physical activity on body composition in overweight and obese adolescents: A longitudinal study differentiating home and school environments
Source: PLoS One. 2025 Feb 26;20(2):e0318000. doi: 10.1371/journal.pone.0318000 (PMC11864506; doi:10.1371/journal.pone.0318000)

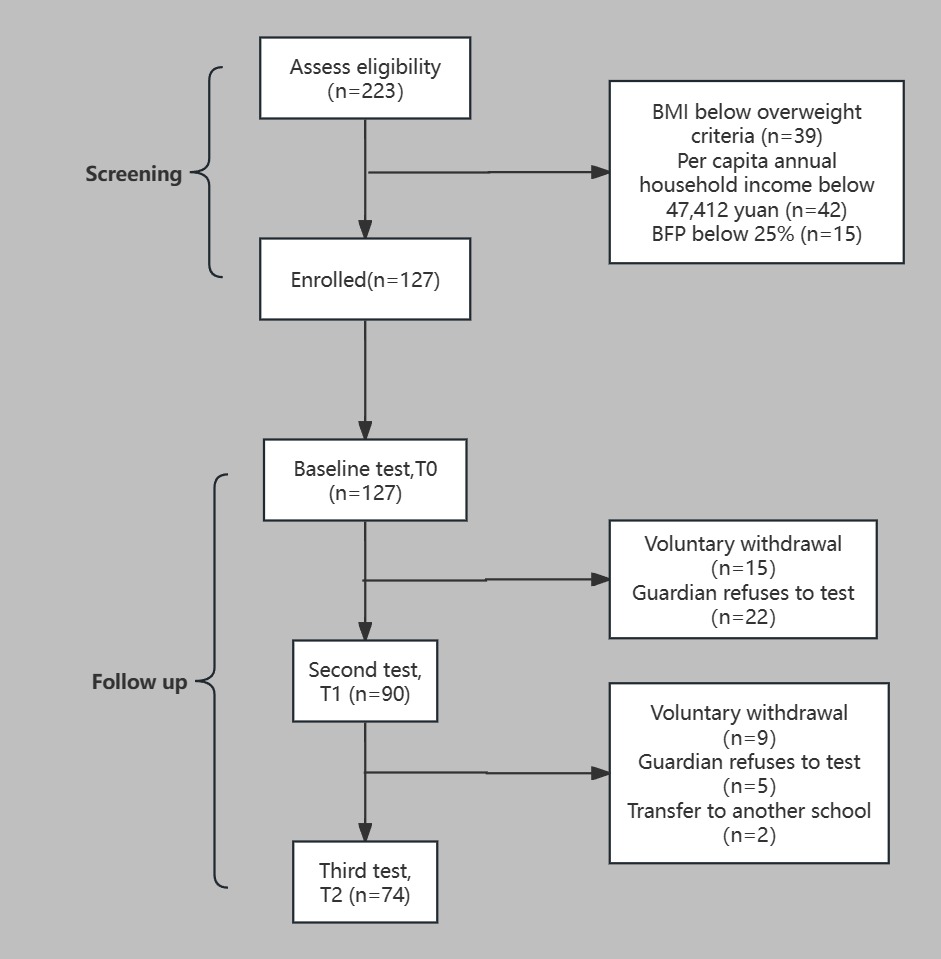

Supplement: S1 Fig — (JPG) [file pone.0318000.s001.jpg]
